# Risk of Necrotizing Enterocolitis Associated With the Single Nucleotide Polymorphisms *VEGF* C-2578A, *IL-18* C-607A, and *IL-4 Receptor* α*-Chain* A-1902G: A Validation Study in a Prospective Multicenter Cohort

**DOI:** 10.3389/fped.2020.00045

**Published:** 2020-02-18

**Authors:** Rob M. Moonen, Maurice J. Huizing, Gema E. González-Luis, Giacomo Cavallaro, Fabio Mosca, Eduardo Villamor

**Affiliations:** ^1^Department of Pediatrics, Zuyderland Medical Center, Heerlen, Netherlands; ^2^Department of Pediatrics, Maastricht University Medical Center (MUMC+), School for Oncology and Developmental Biology (GROW), Maastricht, Netherlands; ^3^Department of Pediatrics, Hospital Universitario Materno-Infantil de Canarias, Las Palmas de Gran Canaria, Spain; ^4^Neonatal Intensive Care Unit, Department of Clinical Sciences and Community Health, Fondazione IRCCS Cà Granda Ospedale Maggiore Policlinico, Università degli Studi di Milano, Milan, Italy

**Keywords:** VEGF, IL-18, IL-4 receptor α-chain, polymorphisms, necrotizing enterocolitis, preterm

## Abstract

The etiology of necrotizing enterocolitis (NEC) is multifactorial and an underlying genetic predisposition to NEC is increasingly being recognized. A growing number of studies identified single nucleotide polymorphisms (SNPs) of selected genes with potential biological relevance in the development of NEC. However, few of these genetic studies have been replicated in validation cohorts. We aimed to confirm in a cohort of 358 preterm newborns (gestational age <30 weeks, 26 cases of NEC ≥ Bell stage II) the association with NEC of three candidate SNPs: the vascular endothelium growth factor (*VEGF*) C-2578A polymorphism (rs699947), the interleukin *(IL)-18* C-607A polymorphism (rs1946518), and the IL-4 receptor α-chain *(IL-4R*α*)* A-1902G polymorphism (rs1801275). We observed that allele and genotype frequencies of the three SNPs did not significantly differ between the infants with and without NEC. In contrast, the minor G-allele of the *IL-4R*α A-1902G polymorphism was significantly less frequent in the group of 51 infants with the combined outcome NEC or death before 34 weeks postmenstrual age than in the infants without the outcome (0.206 vs. 0.331, *P* = 0.01). In addition, a significant negative association of the G-allele with the combined outcome NEC or death was found using the dominant (adjusted odds ratio, aOR: 0.44, 95% CI 0.21–0.92), recessive (aOR 0.15, 95% CI 0.03–0.74), and additive (aOR 0.46, 95% CI 0.26–0.80) genetic models. In conclusion our study provides further evidence that a genetic variant of the *IL-4R*α gene may contribute to NEC.

## Introduction

Necrotizing enterocolitis (NEC) is the leading cause of morbidity and mortality from gastrointestinal disease in very and extremely preterm infants (1, 2). As extensively discussed in several exhaustive reviews, the etiology of NEC is multifactorial and largely related to immaturity of the gastrointestinal tract (1–9). Besides low gestational age (GA), a complex interplay of other factors, such as type of feeding, gut dysbiosis, the high susceptibility of intestinal mucosal surface to inflammatory processes, or intestinal hypoperfusion may contribute to the pathogenesis of NEC (1–9). Nevertheless, NEC affects a minority of (very) preterm infants and clinical risk factors provide a limited explanation of the inter-individual variability in NEC susceptibility (3, 10). In the last years, an underlying genetic predisposition to NEC is increasingly being recognized (3, 10, 11).

As recently reviewed by Cuna et al., the candidate gene approach has been used in most studies on the genetics of NEC (3). Using this approach, a growing number of studies investigated single nucleotide polymorphisms (SNPs) of selected genes based on a-priori hypothesis of relevance to NEC. These studies particularly focused on mediators involved in the regulation of immune/inflammatory responses, such as toll like receptors, interleukins (ILs), tumor necrosis factor, or nuclear factor-kappa beta, and in the control of intestinal microcirculation, such as nitric oxide synthase, or vascular endothelium growth factor (VEGF) (3). However, few of these genetic studies have been replicated in validation cohorts (3). In the present study we aimed to confirm the association with NEC of three candidate SNPs: the *VEGF* C-2578A polymorphism (rs699947) (12, 13), the *IL-18* C-607A polymorphism (rs1946518) (14), and the IL-4 receptor α-chain (*IL-4R*α) A-1902G polymorphism (rs1801275) (15). We performed our investigation in a cohort of preterm infants from four neonatal intensive care units located in three different European countries (Spain, Italy, and the Netherlands) (16, 17).

## Materials and Methods

### Patients

The present study was performed on DNA samples collected during a previous study on carbamoyl phosphate synthetase SNPs as risk factor for NEC (16). The protocol of the above study was reviewed and approved by the institutional review board (IRB) for each participating center (see names below) and we obtained written informed consent from the parents. The consent also allowed, after anonymization of the DNA samples, the investigation of other SNPs potentially involved in NEC development. The protocol is registered in ClinicalTrials.gov Protocol Registration System (NCT00554866). Additional approval was obtained for the present analysis. All infants with a GA ≤30 weeks and birth weight (BW) ≤1,500 g born between July 2007 and July 2012 and admitted to the level III neonatal intensive care unit of the Maastricht University Medical Center (the Netherlands, IRB number MEC-07-2-018), Hospital Universitario Materno-Infantil de Canarias (Las Palmas de Gran Canaria, Spain, IRB number CEIC- 276), Carlo Poma Hospital (Mantova, Italy, inclusion until December 2011, IRB number 21366/2007), and Ospedale Maggiore Policlinico (Milan, Italy, inclusion from January 2012, IRB number 220211b) were eligible for participation in the study. As reported in a previous study, buccal cell samples were obtained from 96 healthy term infants (25 in Maastricht, 31 in Las Palmas de Gran Canaria and 40 in Mantova) (18).

### Definition of Clinical Characteristics and Outcomes

Definitions were identical to those used in our previous study (16) and are therefore literally reproduced here. Data on clinical characteristics and outcomes were obtained from the medical records. GA was determined by the last menstrual period and early ultrasounds (before 20 weeks of gestation). Small for GA was defined as BW for GA below the sex-specific 10th percentile. Chorioamnionitis was defined as every clinical suspicion of infection of the chorion, amnion, amniotic fluid, placenta, or a combination as judged by the obstetrician. Prolonged rupture of membranes was defined as rupture of membranes >24 h before delivery. Prenatal exposure to a single course of antenatal steroids was defined as two doses of betamethasone administered 24 h apart and exposure to a partial course of antenatal steroids was defined as administration of a single dose of betamethasone <24 h prior to delivery.

NEC was defined as Bell stage II disease or greater. Cases of NEC stage III were also separately analyzed. At the conclusion of the study, all cases of NEC were reviewed in a blinded fashion by a panel of 4 investigators of the study. Cases of spontaneous intestinal perforation (i.e., without pathologic evidence of NEC) were excluded from the NEC group. The control group for NEC was formed by the infants without the condition who survived until 34 weeks postmenstrual age (PMA). In another analysis, we investigated the combined outcome NEC or death before 34 weeks PMA.

Arterial hypotension was defined as the need for volume expansion or inotropic support. A diagnosis of sepsis required signs of generalized infection, a positive blood culture, and antibiotic therapy. Respiratory distress syndrome (RDS) was defined as requirement for oxygen supplementation or respiratory support due to tachypnea, grunting, nasal flaring, retractions, or cyanosis. Bronchopulmonary dysplasia (BPD) was defined as a supplemental oxygen requirement at 36 weeks PMA to maintain oxygen saturation >90% (19). Retinopathy of prematurity (ROP) was defined as stage II or higher. Persistent ductus arteriosus (PDA) was defined as a requirement for indomethacin or ibuprofen and/or surgical ligation. Intraventricular hemorrhage (IVH) was classified by using the 4-level grading system (20). Grade <2 IVHs were not included in the analysis.

### Samples and Genotyping

Buccal cell samples for DNA testing were obtained with a sterile OmniSwab (Whatman) and collected in Eppendorf sterile PCR tubes and stored at −80°C until further analysis. The samples obtained in Spain and Italy were transported on dry ice to Maastricht where all the analyses were performed. DNA was extracted using standard methods and stored at −20°C until genotyping. Discrimination of the *VEGF* rs699947, the *IL-18* rs1946518, and the *IL-4R*α rs1801275 alleles was performed with TaqMan® SNP genotyping assays (Applied Biosystems, Foster City, CA, USA) ID C_8311602_10 (rs699947), C_2898460_10 (rs1946518), and C_2351160_20 (rs1801275), following the manufacturer's instructions. The TaqMan technique combines DNA amplification and genotype detection in a single assay (21). Probe sequences are depicted in Supplementary Table 1. Genotyping assays were performed on an ABI PRISM 7900 Sequence Detection System for allelic discrimination (Applied Biosystems).

### Statistical Analysis

Methods for statistical analysis were identical to those used in our previous study (16) and are therefore literally reproduced here. Categorical variables were expressed as counts or percentages and compared using the chi-square test. Continuous variables were expressed as mean (SD) if they followed a normal distribution and compared using unpaired, two-sided *t*-test. If not normally distributed, continuous variables were expressed as median values (interquartile range, IQR; 25th−75th percentile) and compared using the using the Mann–Whitney *U*-test. The Kolmogorov-Smirnov test was used to test for normal distribution of continuous data.

Differences in allelic frequencies and genotype distributions between the investigated populations, as well as Hardy–Weinberg equilibrium (HWE) for genotype distribution were assessed using a chi-square test. The Hardy–Weinberg law states that q2 + 2pq + p2 = 1, where p and q are allele frequencies in a two-allele system. Logistic regression analysis was used to compute the odds ratios (ORs) and their 95% confidence intervals (CI) for NEC and the combined outcome NEC or death before 34 weeks of PMA based on genotype after accounting for the covariates which were significantly different between the groups and are known risk factors for developing NEC. Different genetic models were used to analyze the effect of the risk allele including the general allelic (multiplicative or codominant model), dominant, recessive, and additive models. Assuming a genetic penetrance parameter γ (γ>1), a multiplicative model indicates that the risk of disease is increased γ-fold with each additional copy of the risk allele; an additive model indicates that risk of disease is increased γ-fold for the genotype with one copy of the risk allele and 2 γ-fold for the genotype with two copies of the risk allele; a common recessive model indicates that two copies of the risk allele are required for a γ-fold increase in disease risk, and a common dominant model indicates that either one or two copies of the risk allele are required for a γ-fold increase in disease risk (22). The major allele was considered as a reference and the interactions were tested in the different models by multivariable logistic regression model. All the statistical analyses were performed using IBM SPSS Statistics for Windows, Version 22.0. (IBM Corporation, Armonk, NY, USA) and conducted at the *P* < 0.05 level of significance.

## Results

### Patient Characteristics

A total number of 358 preterm infants (26 NEC cases) were genotyped for the three SNPs, but genotype of *VEGF* C-2578A SNP failed in 9 infants (1 from the NEC group) and genotype of *IL-18* C-607A SNP failed in 1 infant without NEC. Demographic and clinical characteristics of the infants with and without NEC are shown and compared in Table 1. Mean GA and mean BW of infants with NEC were significantly lower than in infants without NEC. In addition, infants with NEC showed a higher incidence of vaginal delivery, mechanical ventilation, BPD, hypotension, IVH, ROP, and mortality. We adjusted for GA, BW, mechanical ventilation, hypotension, and death before 34 weeks PMA in the subsequent logistic regression analysis.

**Table 1:** Baseline characteristics and neonatal complications in preterm infants with and without NEC.

	**NEC-yes** **(*n* = 26)**	***n* data** **missing**	**NEC-no** **(*n* = 332)**	***n* data** **missing**	***P*-value**
Birth weight (g)	873 (SD 230)	0	1,040 (SD 259)	0	0.002
Gestational age (weeks)	26.8 (SD 1.9)	0	28.1 (SD 1.8)	0	0.000
Male sex	11 (42.3)	0	191 (57.5)	0	0.132
Prenatal steroids	18 (72)	1	278 (87.4)	14	0.097
Preecclampsia	2 (8.0)	1	52 (15.9)	5	0.293
Chorioamnionitis	0 (0.0)	0	35 (10.6)	3	0.080
PROM	6 (23.1)	0	95 (29.1)	5	0.516
Vaginal delivery	17 (65.4)	0	147 (44.5)	2	0.040
Apgar (1 min)	5 [3–8]	1	6 [4–8]	3	0.184
Apgar (5 min)	8 [6–9]	1	8 [7–9]	4	0.161
RDS	22 (84.6)	0	274 (82.8)	1	0.811
Mechanical vent.	23 (88.5)	0	202 (61.8)	5	0.006
BPD	15 (57.7)	0	92 (27.9)	2	0.001
Hypotension	19 (73.1)	0	118 (36.0)	4	0.000
Sepsis	17 (70.8)	2	174 (52.7)	2	0.086
IVH	14 (53.8)	0	93 (28.3)	3	0.006
PVL	3 (11.5)	0	13 (4.0)	4	0.073
PDA	15 (60.0)	1	140 (42.6)	3	0.090
ROP	8 (38.1)	5	51 (16.7)	27	0.014
Mortality	8 (30.8)	0	30 (9.0)	0	0.001
Death before 34 weeks	5 (19.2)	0	25 (7.5)	0	0.038

*Results are expressed as mean (SD), median [interquartile range] or absolute numbers of patients (percentage). NEC, necrotizing enterocolitis (≥stage II); PROM, prolonged rupture of membranes; RDS, respiratory distress syndrome; BPD, bronchopulmonary dysplasia; IVH, intraventricular hemorrhage (≥grade 2); PVL, periventricular leukomalacia; PDA, patent ductus arteriosus; ROP, retinopathy of prematurity (≥stage II)*.

The demographic and clinical characteristics of the infants with and without the combined outcome NEC or death before 34 weeks PMA are shown and compared in Table 2. Mean GA, mean BW and median Apgar score at 1 and 5 min of infants with NEC or death were significantly lower than in infants without the combined outcome. In addition, infants with the combined outcome NEC or death showed a higher incidence of vaginal delivery, mechanical ventilation, hypotension, IVH, and PDA. We adjusted for GA, BW, Apgar score after at 1 and 5 min, mechanical ventilation, hypotension, and PDA in the subsequent logistic regression analysis. We did not observe a significant difference between the NEC and the control group in the rate of exposure to antenatal steroids and therefore we did not control for this exposure.

**Table 2:** Baseline characteristics and neonatal complications in preterm infants with and without the combined outcome NEC or death before 34 weeks of corrected gestational age.

	**NEC/death-yes** **(*n* = 51)**	***n* data missing**	**NEC/death-no** **(*n* = 307)**	***n* data missing**	***P*-value**
Birth weight (g)	821 (SD 225)	0	1,063 (SD 250)	0	0.000
Gestational age (weeks)	26.3 (SD 1.9)	0	28.3 (SD 1.6)	0	0.000
Male sex	26 (42.7)	0	176 (57.3)	0	0.397
Prenatal steroids	31 (63.3)	2	259 (88.1)	13	0.060
Preecclampsia	3 (6.0)	1	51 (16.8)	4	0.049
Chorioamnionitis	6 (12.0)	1	29 (9.5)	2	0.584
PROM	15 (30.6)	2	86 (28.3)	3	0.738
Vaginal delivery	32 (62.7)	0	132 (56.7)	2	0.010
Apgar (1 min)	5 [3–6]	1	6 [5-8]	6	0.000
Apgar (5 min)	7 [6–8]	1	8 [7-9]	6	0.000
RDS	43 (84.3)	0	253 (82.7)	1	0.774
Mechanical vent.	48 (94.1)	0	177 (58.6)	5	0.000
BPD	18 (36.0)	1	89 (29.1)	1	0.323
Hypotension	42 (82.4)	0	95 (31.4)	4	0.000
Sepsis	30 (61.2)	2	161 (52.8)	2	0.271
IVH	28 (56.0)	1	79 (25.9)	2	0.000
PVL	4 (8.0)	1	12 (3.9)	3	0.201
PDA	32 (65.3)	2	123 (40.3)	2	0.001
ROP	8 (21.1)	13	51 (17.7)	19	0.615

*Results are expressed as mean (SD), median [interquartile range] or absolute numbers of patients (percentage). NEC, necrotizing enterocolitis (≥stage II); PROM, prolonged rupture of membranes; RDS, respiratory distress syndrome; BPD, bronchopulmonary dysplasia; IVH, intraventricular hemorrhage (≥grade 2); PVL, periventricular leukomalacia; PDA, patent ductus arteriosus; ROP, retinopathy of prematurity (≥stage II)*.

### Analysis of Genotypes

Allele and genotype frequencies of the *VEGF* C-2578A, *IL-18* C-607A, and *IL-4R*α A-1902G polymorphisms in the total preterm population did not significantly differ from the allele and genotype frequencies observed in the population of 96 healthy term infants (Table 3) (18). The distribution of the genotypes of the *VEGF* C-2578A- and the *IL-4R*α A-1902G polymorphisms in the preterm population and the *IL-4R*α A-1902G polymorphism in the term population did not fulfill Hardy-Weinberg criteria (Table 3). The minor allele frequencies (MAFs) of the studied polymorphisms in the NEC ≥ stage II, and NEC stage III groups were not significantly different from the MAFs observed in the infants without NEC (Table 4). The MAF of the *IL-4R*α A-1902G polymorphism was significantly lower (0.206 vs. 0.331, *P* = 0.01) in the group of infants with the combined outcome NEC or death before 34 weeks PMA than in the infants without the outcome (Table 4). The MAFs of the *VEGF* C-2578A, and *IL-18* C-607A SNPS were not significantly different in the group of infants with the combined outcome NEC or death before 34 weeks PMA than in the infants without the outcome (Table 4). The distribution of the genotypes of the *VEGF* C-2578A- and the *IL-4R*α A-1902G polymorphisms in the population of preterm infants without NEC or without the combined outcome NEC/death did not fulfill Hardy-Weinberg criteria (Table 4).

**Table 3:** Genotype characteristics of the *VEGF* C-2578A, *IL-18* C-607A, and *IL-4 receptor* α*-chain* A-1902G polymorphism in total preterm study group (*n* = 358) and term control group (*n* = 96).

	**Chromosomal location**	**Allele W/M**	**Total study group** **WW/WM/MM alleles** **(prevalence of** **mutant allele)**	**Missing data** **(*n*)**	**HWE** **(*P-*value)**	**Term control group** **WW/WM/MM alleles** **(prevalence of** **mutant allele)**	**Missing data** **(*n*)**	**HWE in controls** **(*P-*value)**
*VEGF* C-2578A	6p21.1	C/A	109/155/85 (0.466)	9	0.04^*^	33/47/16 (0.411)	0	0.92
*IL-18* C-607A	11q23.1	C/A	102/177/78 (0.466)	1	0.94	27/48/18 (0.452)	3	0.69
*IL-4 R* A-1902G	16p12.1	A/G	179/134/45 (0.313)	0	0.01^*^	56/29/10 (0.258)	1	0.049^*^

Results are expressed as absolute numbers of patients. Missing data due to technical inability of genotyping sample.

HWE, Hardy–Weinberg Equilibrium; W, wild type allele; M, mutant allele.

* *P < 0.05*.

**Table 4:** Genotype distribution and minor allele frequency of the *VEGF* C-2578A, *IL-18* C-607A, and *IL-4 R* A-1902G polymorphisms for NEC ≥stage II, NEC stage III and the combined outcome NEC or death before 34 weeks of corrected gestational age.

**SNP**	**Genotype**	**NEC-no** ***n* (%)**	**MAF**	**NEC (≥stage II)-yes** ***n* (%)**	**MAF**	**NEC (stage III)-yes *n* (%)**	**MAF**	**NEC/death-no *n* (%)**	**MAF**	**NEC/death-yes** ***n* (%)**	**MAF**
*VEGF* C-2578A	CC	101 (31.2)	0.468	8 (32.0)	0.440	2 (20.0)	0.500	95 (31.3)	0.467	14 (31.1)	0.456
	CA	143 (44.1)		12 (48.0)		6 (60.0)		134 (44.1)		21 (46.7)	
	AA	80 (24.7)^†^		5 (20.0)		2 (20.0)		75 (24.6)^†^		10 (22.2)	
*IL-18* C-607A	CC	96 (29.0)	0.465	6 (23.1)	0.481	5 (45.5)	0.318	85 (27.8)	0.474	17 (33.3)	0.422
	CA	162 (48.9)		15 (57.7)		5 (45.5)		152 (49.7)		25 (49.0)	
	AA	73 (22.1)		5 (19.2)		1 (9.1)		69 (22.5)		9 (17.7)	
*IL-4 R* A-1902G	AA	165 (49.7)	0.318	14 (53.8)	0.250	7 (63.6)	0.227	147 (47.9)	0.331	32 (62.7)	0.206^*^
	AG	123 (37.0)		11 (42.3)		3 (27.3)		117 (38.1)		17 (33.3)	
	GG	44 (13.3)^††^		1 (3.9)		1 (9.1)		43 (14.0)^††^		2 (4.0)	

SNP, single-nucleotide polymorphism; NEC, necrotizing enterocolitis; MAF, minor allele frequency.

CC denotes homozygosity for the C-encoded VEGF C-2578A and IL-18 C-607A polymorphism variant; AA homozygosity for the A-encoded VEGF C-2578A and IL-18 C-607A polymorphism variant; CA heterozygosity for VEGF C-2578A and IL-18 C-607A polymorphism; AA denotes homozygosity for the A-encoded IL4R A-1902G polymorphism variant; GG homozygosity for the G-encoded IL4R A-1902G polymorphism variant; AG heterozygosity for IL4R A-1902G polymorphism.

* P < 0.05 vs. NEC/death-no.

†,†† *P < 0.05, 0.01 for deviation of Hardy-Weinberg equilibrium in the disease-free group*.

We further analyzed the effect of the *VEGF* C-2578A-, *IL-18* C-607A- and *IL-4R*α A-1902G polymorphism on the occurrence of NEC and NEC or death under different genetic models. Logistic regression analysis could not detect any significant association between the studied polymorphisms and NEC ≥ stage II (Table 5), or NEC stage III (Table 6) in any of the genetic models. In contrast, the co-dominant, dominant, recessive, and additive model showed a negative significant association of the G-allele of the *IL-4R*α A-1902G polymorphism with the combined outcome NEC or death before 34 weeks of corrected gestational age (Table 7).

**Table 5:** Effects of *VEGF* C-2578A, *IL-18* C-607A, and *IL-4 R* A-1902G polymorphisms on the risk of NEC (≥ stage II) under different genetic models.

**SNP**	**Genotype**	**Codominant model**				**Dominant model**				**Recessive model**				**Additive model**			
		**OR (95% CI)**	***P*-value**	**aOR (95% CI)**	***P*-value**	**OR (95% CI)**	***P*-value**	**aOR (95% CI)**	***P*-value**	**OR (95% CI)**	***P*-value**	**aOR (95% CI)**	***P*-value**	**OR (95% CI)**	***P*-value**	**aOR (95% CI)**	***P*-value**
*VEGF*	CC	1 (Ref.)	–	1 (Ref.)	–	0.96 (0.40–2.30)	0.93	1.08 (0.44–2.67)	0.86	0.76 (0.28–2.10)	0.60	0.88 (0.31–2.49)	0.80	0.90 (0.52–1.57)	0.72	0.99 (0.56–1.76)	0.98
	CA	1.06 (0.42–2.69)	−0.90	1.15 (0.44–3.01)	0.93												
	AA	0.79 (0.25–2.51)	0.69	0.95 (0.29–3.13)	0.86												
*IL-18*	CC	1 (Ref.)	–	1 (Ref.)	–	1.36 (0.53–3.50)	0.52	1.40 (0.52–3.73)	0.50	0.84 (0.31–2.31)	0.74	0.84 (0.30–2.39)	0.74	1.06 (0.61–1.87)	0.83	1.07 (0.60–1.94)	0.81
	CA	1.48 (0.56–3.95)	0.43	1.52 (0.55–4.23)	0.42												
	AA	1.10 (0.32–3.73)	0.88	1.12 (0.31–3.99)	0.86												
*IL-4 R*	AA	1 (Ref.)	–	1 (Ref.)	–	0.85 (0.38–1.89)	0.68	0.85 (0.36–1.97)	0.70	0.26 (0.04–1.98)	0.19	0.23 (0.03–1.78)	0.16	0.74 (0.40–1.37)	0.34	0.72 (0.38–1.36)	0.31
	AG	1.05 (0.46–2.40)	0.90	1.09 (0.46–2.61)	0.84												
	GG	0.27 (0.03–2.09)	0.21	0.24 (0.03–1.92)	0.18												

SNP, single-nucleotide polymorphism; NEC, necrotizing enterocolitis (≥stage II); MAF, minor allele frequency; OR, odds ratio; aOR, odds ratio adjusted for birth weight, gestational age, mechanical ventilation, hypotension, and death before 34 weeks.

*CC denotes homozygosity for the C-encoded VEGF C-2578A and IL-18 C-607A polymorphism variant; AA homozygosity for the A-encoded VEGF C-2578A and IL-18 C-607A polymorphism variant; CA heterozygosity for VEGF C-2578A and IL-18 C-607A polymorphism; AA denotes homozygosity for the A-encoded IL4R A-1902G polymorphism variant; GG homozygosity for the G-encoded IL4R A-1902G polymorphism variant; AG heterozygosity for IL4R A-1902G polymorphism*.

**Table 6:** Effects of *VEGF* C-2578A, *IL-18* C-607A, and *IL-4 R* A-1902G polymorphisms on the risk of NEC stage III under different genetic models.

**SNP**	**Genotype**	**Codominant model**				**Dominant model**				**Recessive model**				**Additive model**			
		**OR (95% CI)**	***P*-value**	**aOR (95% CI)**	***P*-value**	**OR (95% CI)**	***P*-value**	**aOR (95% CI)**	***P*-value**	**OR (95% CI)**	***P*-value**	**aOR (95% CI)**	***P*-value**	**OR (95% CI)**	***P*-value**	**aOR (95% CI)**	***P*-value**
*VEGF*	CC	1 (Ref.)	–	1 (Ref.)	–	1.81 (0.38–8.68)	0.46	2.76 (0.51–14.9)	0.24	0.76 (0.16–3.67)	0.74	1.20 (0.21–6.74)	0.84	1.13 (0.48–2.62)	0.79	1.56 (0.60–4.08)	0.36
	CA	2.12 (0.42–10.7)	0.36	2.93 (0.51–16.9)	0.23												
	AA	1.26 (0.17–9.16)	0.82	2.38 (0.28–20.2)	0.43												
*IL-18*	CC	1 (Ref.)	–	1 (Ref.)	–	0.49 (0.15–1.64)	0.25	0.44 (0.12–1.69)	0.23	0.35 (0.05–2.81)	0.33	0.26 (0.03–2.43)	0.24	0.54 (0.22–1.34)	0.18	0.48 (0.18–1.28)	0.14
	CA	0.59 (0.17–2.10)	0.42	0.57 (0.14–2.31)	0.43												
	AA	0.26 (0.03–2.30)	0.23	0.19 (0.02–1.99)	0.34												
*IL-4 R*	AA	1 (Ref.)	–	1 (Ref.)	–	0.57 (0.16–1.97)	0.37	0.72 (0.18–2.82)	0.64	0.66 (0.08–5.24)	0.69	0.65 (0.07–5.91)	0.70	0.67 (0.26–1.73)	0.41	0.77 (0.29–2.08)	0.61
	AG	0.58 (0.15–2.27)	0.43	0.77 (0.17–3.50)	0.74												
	GG	0.54 (0.06–4.47)	0.56	0.60 (0.06–5.69)	0.65												

SNP, single-nucleotide polymorphism; NEC, necrotizing enterocolitis (stage III); MAF, minor allele frequency; OR, odds ratio; aOR, odds ratio adjusted for weight, gestational age, mechanical ventilation, hypotension, and death before 34 weeks.

*CC denotes homozygosity for the C-encoded VEGF C-2578A and IL-18 C-607A polymorphism variant; AA homozygosity for the A-encoded VEGF C-2578A and IL-18 C-607A polymorphism variant; CA heterozygosity for VEGF C-2578A and IL-18 C-607A polymorphism; AA denotes homozygosity for the A-encoded IL4R A-1902G polymorphism variant; GG homozygosity for the G-encoded IL4R A-1902G polymorphism variant; AG heterozygosity for IL4R A-1902G polymorphism*.

**Table 7:** Effects of *VEGF* C-2578A, *IL-18* C-607A and *IL-4 R* A-1902G polymorphisms on the risk of the combined outcome NEC or death before 34 weeks of corrected gestational age under different genetic models.

**SNP**	**Genotype**	**Codominant model**				**Dominant model**				**Recessive model**				**Additive model**			
		**OR (95% CI)**	***P*-value**	**aOR (95% CI)**	***P*-value**	**OR (95% CI)**	***P*-value**	**aOR (95% CI)**	***P*-value**	**OR (95% CI)**	***P*-value**	**aOR (95% CI)**	***P*-value**	**OR (95% CI)**	***P*-value**	**aOR (95% CI)**	***P*-value**
*VEGF*	CC	1 (Ref.)	–	1 (Ref.)	–	1.01 (0.51–1.98)	0.99	1.34 (0.58–3.09)	0.50	0.87 (0.41–1.85)	0.72	1.24 (0.51–3.02)	0.63	0.96 (0.63–1.46)	0.85	1.21 (0.72–2.02)	0.48
	CA	1.06 (0.52–2.20)	0.87	1.29 (0.52–3.17)	0.59												
	AA	0.91 (0.38–2.15)	0.82	1.44 (0.51–4.11)	0.49												
*IL-18*	CC	1 (Ref.)	–	1 (Ref.)	–	0.77 (0.41–1.45)	0.42	0.73 (0.34–1.56)	0.41	0.74 (0.34–1.59)	0.43	0.66 (0.26–1.65)	0.37	0.81 (0.53–1.24)	0.33	0.77 (0.46–1.27)	0.30
	CA	0.82 (0.42–1.61)	0.57	0.80 (0.36–1.80)	0.59												
	AA	0.65 (0.27–1.55)	0.34	0.58 (0.21–1.63)	0.30												
*IL-4 R*	AA	1 (Ref.)	–	1 (Ref.)	–	0.55 (0.30–1.00)	0.05	0.44 (0.21–0.92)	0.03^*^	0.25 (0.06–1.07)	0.06	0.15 (0.03–0.74)	0.02^*^	0.56 (0.34–0.91)	0.02^*^	0.46 (0.26–0.80)	0.01^*^
	AG	0.68 (0.35–1.26)	0.21	0.61 (0.28–1.32)	0.21												
	GG	0.21 (0.05–0.93)	0.04^*^	0.13 (0.03–0.63)	0.01^*^												

SNP, single-nucleotide polymorphism; NEC, necrotizing enterocolitis (≥stage II); MAF, minor allele frequency; OR, odds ratio; aOR, odds ratio adjusted for birth weight, gestational age, Apgar score at 1 min, Apgar score at 5 min, mechanical ventilation, hypotension and PDA.

CC denotes homozygosity for the C-encoded VEGF C-2578A and IL-18 C-607A polymorphism variant; AA homozygosity for the A-encoded VEGF C-2578A and IL-18 C-607A polymorphism variant; CA heterozygosity for VEGF C-2578A and IL-18 C-607A polymorphism; AA denotes homozygosity for the A-encoded IL4R A-1902G polymorphism variant; GG homozygosity for the G-encoded IL4R A-1902G polymorphism variant; AG heterozygosity for IL4R A-1902G polymorphism.

* *P < 0.05*.

## Discussion

Given that NEC remains a leading cause of morbidity and mortality, identifying preterm infants at increased risk for developing the condition remains an important but elusive objective. Previous studies suggested an association between the risk of NEC and the SNPs *VEGF* C-2578A, *IL-18* C-607A (14), and *IL-4R*α A-1902G (15). In the present cohort we could not confirm the association between the *VEGF* C-2578A and *IL-18* C-607A SNPs and the risk of NEC or the combined outcome NEC or death before 34 weeks of corrected gestation. However, the co-dominant, dominant, recessive, and additive models showed a significant negative association of the G-allele of the *IL-4R*α A-1902G polymorphism with the combined outcome NEC or death before 34 weeks of corrected gestation.

This study has some limitations that need to be considered. First, we did not investigate the functional consequences of the SNPs. Second, the distribution of the genotypes of the *VEGF* C-2578A- and the *IL-4R*α A-1902G polymorphisms in the preterm control population and the *IL-4R*α A-1902G polymorphism in the term population did not fulfill Hardy-Weinberg criteria. Theoretically, disease-free control groups from outbred populations should not deviate from HWE (23). Deviation from HWE may be related to non-random mating, population stratification, selection bias, limited sample size, or genotyping error (23). Of note, deviation from HWE in the control group may increase the chance of detecting a false-positive association, particularly when ORs did not show a strong association (23). This points to the need for caution in interpreting our findings.

VEGF is an angiogenic protein that couples hypoxia sensing to angiogenesis and is necessary for the development and maintenance of capillary networks (9). It is suggested that VEGF-mediated alterations of the intestinal mucosal microvasculature play an important role in NEC pathogenesis (9, 13, 24, 25). In fact, intestinal VEGF protein expression is reduced in human and experimental NEC (9, 26). The *VEGF* gene is highly polymorphic, especially in the promoter, 5′-untranslated and 3′-untranslated regions (27). Some of these SNPs, including the C-2578A, have been related to varying VEGF protein expression and serum VEGFA levels and proposed to be involved in the pathogenesis of various adult diseases (27–30).

As mentioned in the introduction, two previous studies showed that the A allele of the *VEGF* C-2578A SNP increased the risk of NEC in preterm infants (12, 13). Moreover, one of the studies showed that plasma VEGFA levels were significantly lower in carriers of the A allele (13). In contrast, our study could not confirm the association between the *VEGF* C-2578A SNP and NEC. Differences in the inclusion criteria and the ethnic background of the studied populations may account for these different results. The study of Banyasz et al. included infants with all stages of NEC (12), whereas our study and the study of Gao et al. (13) only included infants with confirmed NEC (Bell stage II disease or greater). As highlighted by Cuna et al., a precise phenotypic definition of NEC is essential in the design of genetic studies and only confirmed cases of NEC in preterm infants should be included. The study of Gao et al. (13) involved a Chinese Han population and a simple association may not translate to a population of different ethnic backgrounds (31). An important point of consideration is that the Chinese population has a lower prevalence of the A allele of the *VEGF* C-2578A SNP (32–34) than the Caucasian population (28).

Excessive inflammation is a hallmark in the pathogenesis of NEC and, therefore, many investigators have examined the potential association between several SNPs of pro-and anti-inflammatory cytokine genes and NEC (3, 10). Altered IL-18 levels are present in patients with inflammatory bowel disease (35) and IL-18 deficient mice are showed decreased intestinal damage following experimental NEC (36). Alterations of IL-18 production have been associated with the C-607A SNP of the *IL-18* gene (37). Heninger et al. showed in a case-control studies including 136 preterm newborns (46 NEC, 90 control) that the frequency of the AA genotype of the C-607A SNP was higher in infants with stage III NEC compared to those with stages I–II and those without NEC (14). We could not replicate their results in our cohort.

IL-4 is another cytokine with powerful anti-inflammatory actions. Th1-cell proliferation is inhibited by IL-4 and IL-4 opposes the effects of pro-inflammatory cytokines on macrophages. Isolated lamina propria mononuclear cells from inflamed intestine expressed IL-4 mRNA and secreted this cytokine in lower amounts than control cells (38, 39). The *IL-4R*α A-1902G polymorphism has been shown to affect receptor signaling and the anti-inflammatory effect of IL-4 is more pronounced in peripheral blood mononuclear cells from adults carrying the G-variant of the SNP (40). Trezl et al. showed that carriers of the mutant allele of the *IL-4R*α A-1902G polymorphism had a lower risk of all stages of NEC (15). In contrast, our study could not demonstrate an association between the *IL-4R*α A-1902G SNP and the risk of NEC (≥ Bell stage II). Nevertheless, we found a significant negative association of the G-allele of the *IL-4R*α A-1902G polymorphism with the combined outcome NEC or death before 34 weeks of corrected gestation in all genetic models. We analyzed this combined outcome because epidemiological studies indicate that the time to onset of NEC follows a bimodal distribution with a first peak around at 1 week of age and a second peak around at the end of the third week of life (41, 42). Therefore, early death is a competing outcome for NEC.

As reported by Treszl et al. (15, 38), the *IL-4R*α G1902 variant does not influence IL-4 levels, but its presence is associated with enhanced transduction of IL-4 signals. This enhanced IL-4 transduction is known to shift the development of lymphocytes to a more pronounced Th2 proliferation. They speculate that the elevated number of Th2 cells in carriers of this genetic polymorphism is a protective factor against the development of NEC (15, 38). Accordingly, our data suggest that the minor G variant of the IL-4RαA-1902G SNP might protect against the combined outcome NEC/early death. However, there is still a need for further studies to determine the mechanisms of the protective role of the *IL-4R*α G1902 variant in NEC.

## Conclusion

This is one of the largest cohort studies investigating the association of SNPs involved in the homeostasis of intestinal microcirculation and inflammatory response with NEC. We observed that the *IL-4R*α A-1902G genotype was associated with the risk of developing the combined outcome of NEC or death before 34 weeks PMA. Nevertheless, NEC is a condition with a complex and multifactorial pathogenesis and, therefore, an isolated genetic derangement may not be sufficient to account for the entire complexity of the disease. Many genetic variations associated to prematurity, intestinal function, microcirculatory regulation, inflammatory signaling, or immune defense could protect or increase the susceptibility of preterm infants to NEC (3, 43). The future challenge resides in identifying the haplotypes that confer increased risk for NEC and in understanding their interaction with environmental risk factors. In addition, further investigation is required to determine functional consequences of each SNP.

## Data Availability Statement

This article contains previously unpublished data. Data are available upon request to the authors.

## Ethics Statement

The present study was performed on DNA samples collected during a previous study on carbamoyl phosphate synthetase SNPs as risk factor for NEC. The protocol of the above study was reviewed and approved by the Research Ethics Committees of the participating centers and we obtained written informed consent from the parents. The protocol is registered in ClinicalTrials.gov Protocol Registration System (NCT00554866). Additional approval was obtained for the present analysis.

## Author Contributions

RM participated in the design of the study, collection of data, analysis and interpretation of data, and drafted the first version of the manuscript. MH, GG-L, GC, and FM collected data, participated in the design of the study and the interpretation of the data, and helped to draft the manuscript. EV conceived the study, participated in the design of the study, collection of data, analysis and interpretation of data, and drafted the final version of the manuscript. All authors read and approved the final manuscript.

### Conflict of Interest

The authors declare that the research was conducted in the absence of any commercial or financial relationships that could be construed as a potential conflict of interest.
