# Supplementary material for: Risk of Necrotizing Enterocolitis Associated With the Single Nucleotide Polymorphisms VEGF C-2578A, IL-18 C-607A, and IL-4 Receptor α-Chain A-1902G: A Validation Study in a Prospective Multicenter Cohort
Source: Front Pediatr. 2020 Feb 18;8:45. doi: 10.3389/fped.2020.00045 (PMC7039854; doi:10.3389/fped.2020.00045)
Supplement: Supplementary file 1 [file Data_Sheet_1.PDF]

Supplementary Material

Risk of necrotizing enterocolitis associated with the single nucleotide polymorphisms *VEGF* C-2578A, *IL-18* C-607A, and *IL-4 receptor α-chain* A-1902G: A validation study in a prospective multicenter cohort

Rob M. Moonen[1,2], Maurice J. Huizing[2], Gema E. González-Luis[3], Giacomo Cavallaro[4], Fabio Mosca[4], Eduardo Villamor[2]

[1] Department of Pediatrics, Zuyderland Medical Center, Heerlen, the Netherlands; Email: ro.moonen@zuyderland.nl
[2] Department of Pediatrics, Maastricht University Medical Center (MUMC+), School for Oncology and Developmental Biology (GROW), Maastricht, the Netherlands; Emails: m.huizing@mumc.nl; e.villamor@mumc.nl
[3] Department of Pediatrics, Hospital Universitario Materno-Infantil de Canarias, Las Palmas de Gran Canaria, 35016, Spain; Email: ggonlui@gobiernodecanarias.org
[4] Neonatal Intensive Care Unit, Department of Clinical Sciences and Community Health, Fondazione IRCCS Cà Granda Ospedale Maggiore Policlinico, Università degli Studi di Milano, Milan, 20122, Italy; Emails: giacomo.cavallaro@mangiagalli.it; fabio.mosca@mangiagalli.it

Correspondence:
Eduardo Villamor
Department of Pediatrics, Maastricht University Medical Center (MUMC+), School for Oncology and Developmental Biology (GROW), Maastricht, the Netherlands
e.villamor@mumc.nl

1 Supplementary Tables

Supplementary Table 1. TaqMan® probes used for polymorphism genotyping

Polymorphism	TaqMan® probe sequence [VIC/FAM]
VEGF C-2578A (rs699947)	GCCAGCTGTAGGCCAGACCCTGGCA[A/C]GATCTGGGTGGATAATCAGACTGAC
IL-18 C-607A (rs1946518)	ACGGATACCATCATTAGAATTTTAT[G/T]TAATAATTTTTACACTTTCTGCAAC
IL-4Rα A-1902G (rs1801275)	GTCTCGGCCCCCACCAGTGGCTATC[A/G]GGAGTTTGTACATGCGGTGGAGCAG
